# Supplementary material for: Physiological Characteristics of Sunburn Peel after Apple Debagged
Source: Molecules. 2022 Jun 11;27(12):3775. doi: 10.3390/molecules27123775 (PMC9229340; doi:10.3390/molecules27123775)
Supplement: Supplementary file 1 [file molecules-27-03775-s001.zip › molecules-1738573-supplementary.pdf]

Physiological characteristics of sunburn peel after apple debagged

Yifeng Feng [1], Shanshan Li [1], Rongjian Jia [1], Jie Yang [1], Qiufang Su [1] and Zhengyang Zhao [1,*]

Supplementary

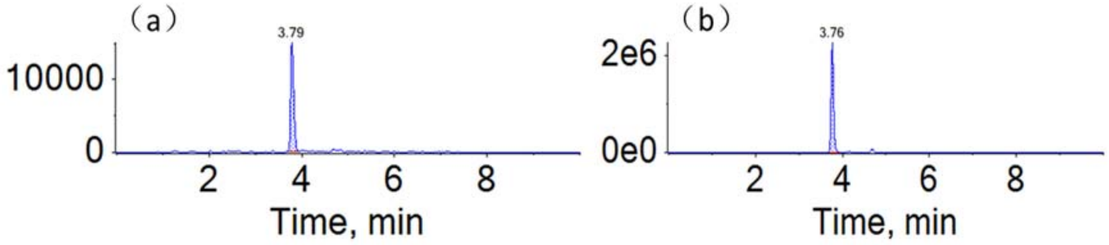

（1）Rutin standard（a） and sample（b） chromatograms

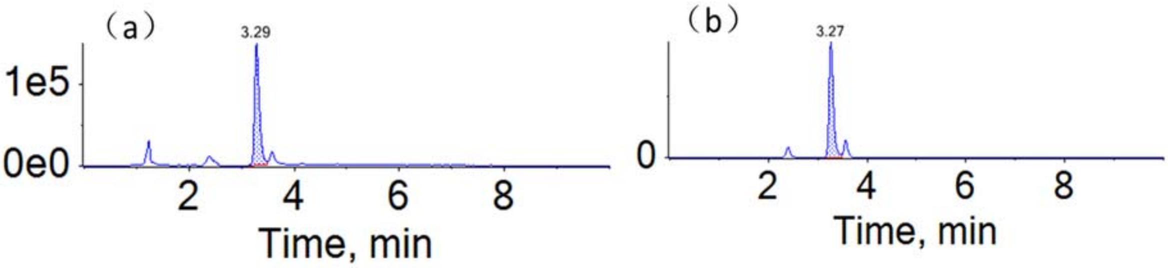

（2）Chlorogenic acid standard（a） and sample（b） chromatograms

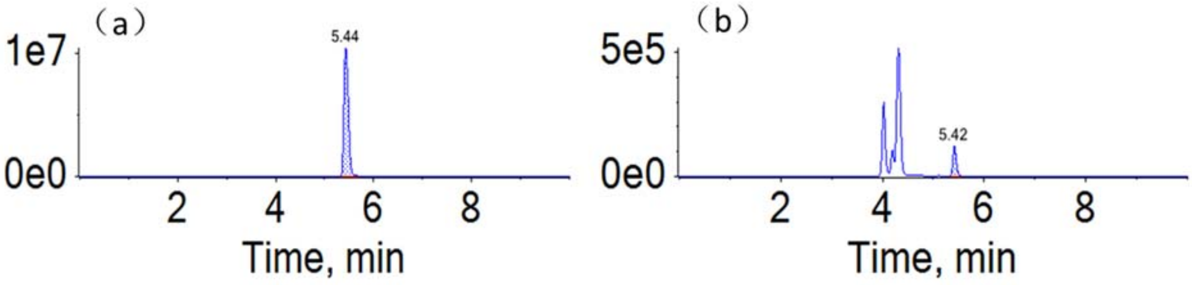

（3）Isoquercitrin standard（a） and sample（b） chromatograms

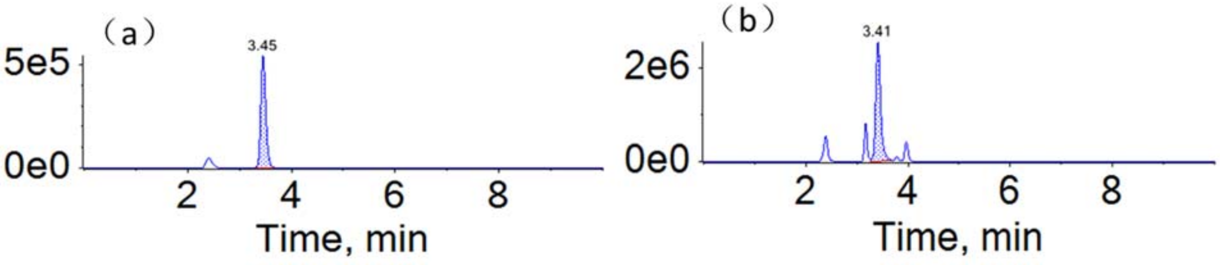

（4）Procyanidin B2 standard（a） and sample（b） chromatograms

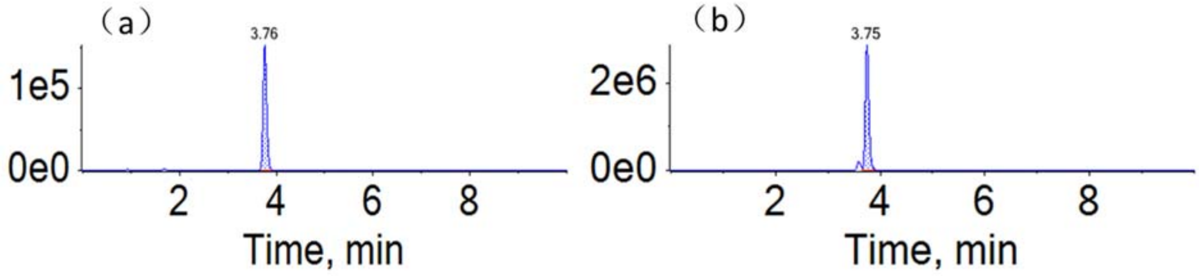

（5）Epicatechin standard（a） and sample（b） chromatograms

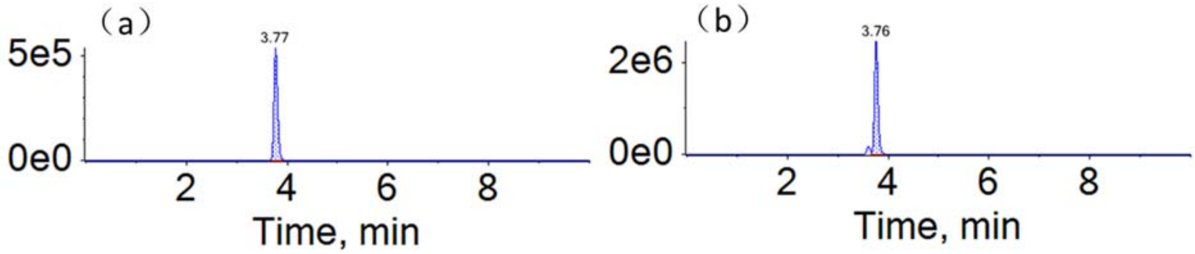

（6）Catechinic acid standard（a） and sample（b） chromatograms

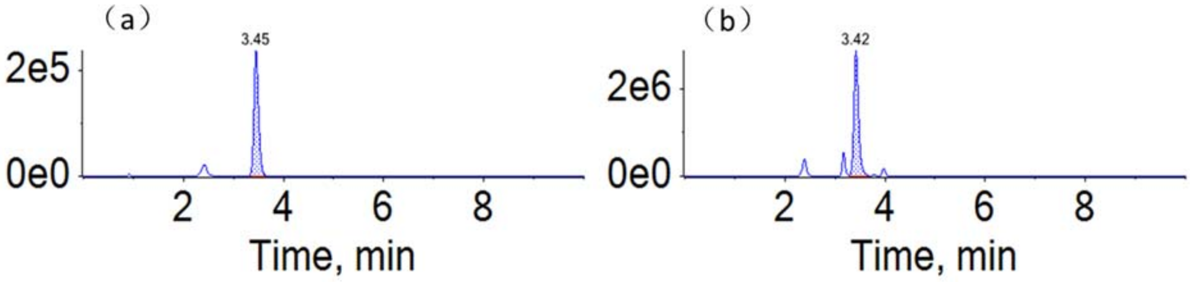

（7）Procyanidin B1 standard（a） and sample（b） chromatograms

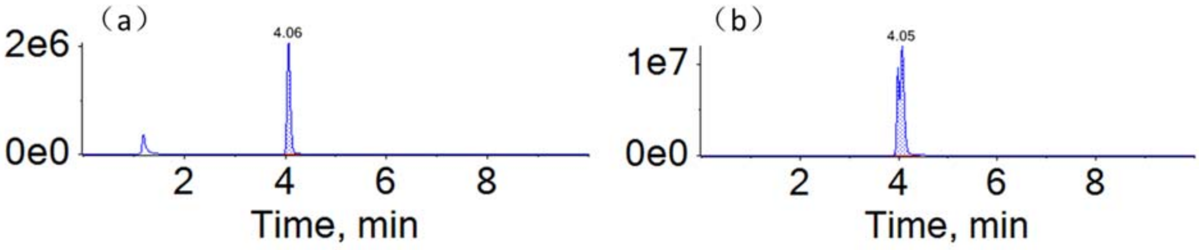

（8）Hyperoside standard（a） and sample（b） chromatograms

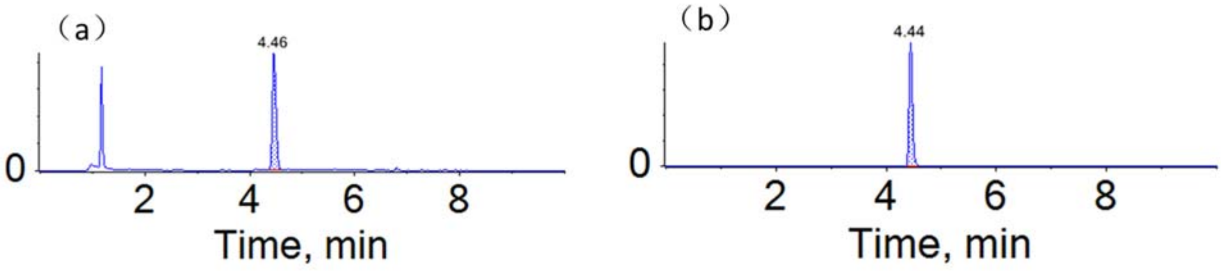

（9）Phloridzin standard（a） and sample（b） chromatograms

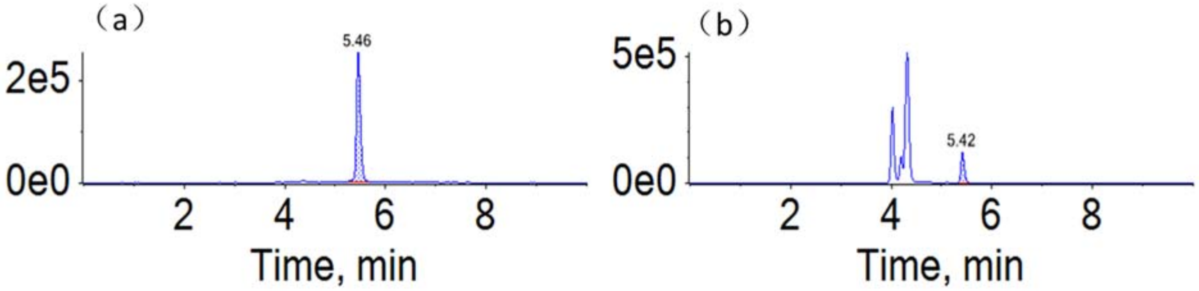

（10）Quercetin standard（a） and sample（b） chromatograms

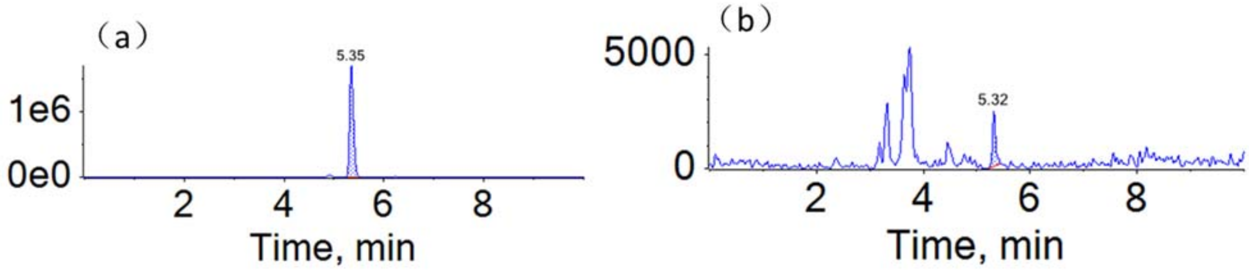

（11）Ferulic acid standard（a） and sample（b） chromatograms

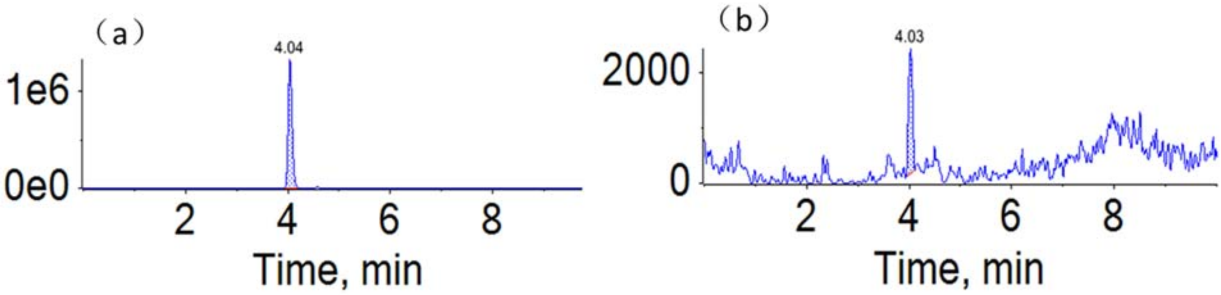

（12）Methyl gallate standard（a） and sample（b） chromatograms

Schedule S1 The chromatogram of standard and samples
